# Treatment of HeartMate III–LVAD driveline infection by negative pressure wound therapy: Result of our case series

**DOI:** 10.1177/03913988211047250

**Published:** 2021-09-24

**Authors:** Mustafa Cikirikcioglu, Kevin Ponchant, Nicolas Murith, Philippe Meyer, Nurcan Yilmaz, Christoph Huber

**Affiliations:** 1 Division of Cardiovascular Surgery, Department of Surgery, University Hospitals and Faculty of Medicine, Geneva, Switzerland; 2 Division of Cardiology, Department of Internal Medicine, University Hospitals and Faculty of Medicine, Geneva, Switzerland

**Keywords:** Device-related infection, left ventricular assist devices, driveline infections, heart failure, negative pressure wound therapy, HeartMate III

## Abstract

Driveline infection is one of the most frequent complications following left ventricular assist device (LVAD) treatment and there is no consensus for its management. The standard approach to treat foreign-body infection is complete device ablation, which is not always feasible and therefore not an elected method for LVAD driveline infections. Here we share the results from a series of cases successfully treated for driveline infection by negative pressure wound therapy (NPWT) therapy. Between 2016 and 2020, five male patients were hospitalized in our unit with a driveline infection of HeartMate III-LVAD^®^. Ultrasonography and/or thoraco-abdominal CT confirmed the diagnosis, infection localization, and abscess formation. Following an antibiotic treatment, an urgent surgical abscess drainage and debridement of the infected tissues were performed. At the end of the procedure, NPWT was applied. NPWT re-dressing and debridement of wound was performed every 3–4 days. The wound was closed surgically after obtaining negative culture results and good healing. The patients were discharged in good condition, without signs of infection. Two patients underwent successful heart transplantation after 1 and 13 months. Other patients did not show any residual or recurrent infection during the follow-up within 25 months. Driveline infection following LVAD implantation is a significant complication and a challenging in terms of management for both; the surgical team and the patient. These results from our case series report a successful and less invasive approach by using NPWT for the treatment of LVAD driveline infections.

## Introduction

The gold standard treatment of heart failure refractory to maximal medical management and conventional surgery consists on heart transplantation, however, the demand for organs exceeds the supply.^1^ For this particular reason, the development of left ventricular assist device (LVAD) has emerged.^2^ Despite significant advances in technology, managing the energy supply to LVAD continues to be faced with challenges. Indeed, due to the requirement of an external power source mediated by a percutaneous tunneled driveline, the device can be constraining and convey high risk of complications, for example driveline infection.^3^ Driveline infection (DLI) is one of the most frequent LVAD complications and a consensus about its management has yet to be determined.^4,5^ In this article, we present a series of five patients suffering from DLI, successfully treated by negative pressure wound therapy (NPWT).

## Patients and methods

Between 2015 and 2020, 20 patients underwent HeartMate II^®^ (one patient) and HeartMate III^®^ (19 patients, all male) LVAD implantation in our institution. In these 20 patients, five male patients (25%) (median age 56—min: 44 max: 71 years old) were hospitalized with DLI of HeartMate III-LVAD^®^.

Patients etiologies responsible for LVAD implantation, ranged from idiopathic dilated cardiomyopathies (*n* = 2), non-compaction associated with valvular cardiomyopathies (*n* = 1), ischemic cardiomyopathies (*n* = 2). Indications for LVAD implantation included destination therapy (*n* = 1) and four bridges to heart transplantation (*n* = 4).

**Figure 1.**
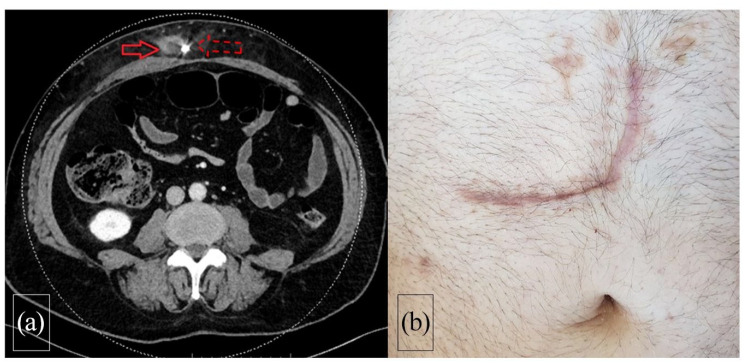
(a) Thoraco-abdominal CT shows important abscess (solid arrow) along the LVAD driveline (dashed arrow) in the abdominal wall and (b) follow-up of the patient following NPWT and surgical closure shows no sign of infection.

**Figure 2.**
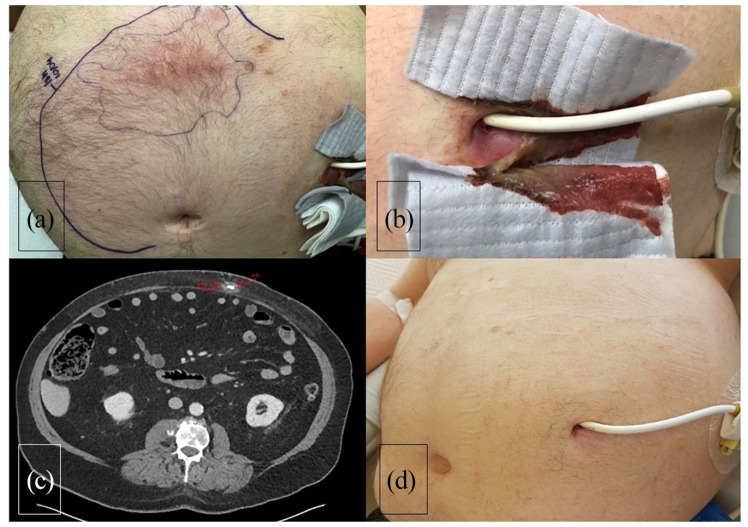
(a) Thoraco-abdominal CT shows infiltration and lobulated liquid collection (solid arrow) along the LVAD driveline (dashed arrow) in the abdominal wall, (b) erythema on the patient skin over the driveline route, (c) abundant purulent discharge through the driveline exit, and (d) follow-up of the patient following NPWT and surgical closure shows no sign of infection.

**Figure 3.**
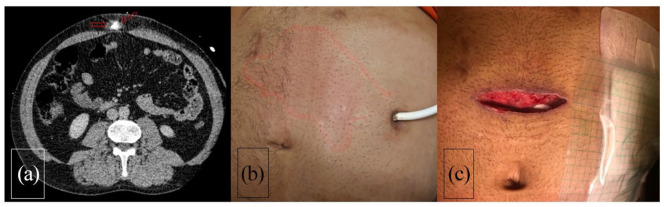
(a) Thoraco-abdominal CT shows abscess (solid arrow) along the LVAD driveline (dashed arrow) in the abdominal wall, (b) erythema on the patient skin over the driveline route, and (c) local state of the infection site before the closure of the NPWT pocket.

The occurrence time of DLI post-implantation oscillated between 4 and 16 months (median: 13 months). The symptoms manifested as either a serosanguinous or a purulent discharge from the exit orifice of the driveline, cutaneous erythema, pain on driveline parietal trajectory, and high fever (Figures 1–3). Blood count exams demonstrated leukocytosis and high CRP.

Diagnosis, localization of the infection and abscess formation were confirmed by ultrasonography and/or thoracoabdominal Computed Tomography (Figures 1–3).

In all cases, the infection was located only around the driveline without reaching the LVAD. Pathogens were mainly Methicillin Sensitive Staphylococcus Aureus (MSSA). Antibiotic therapy varied between patients, and is summarized in Table 1. Following an antibiotic treatment, an urgent surgical drainage of abscess and extensive debridement of infected tissue was performed to obtain healthy tissue. At the end of the procedure, NPWT was applied. The duration of NPWT was between 5 and 16 days (median: 9 days) with one NPWT redressing on average. NPWT re-dressing and wound debridement was performed every 4–5 days. The wound was closed surgically with simple resorbable stiches for subcutaneous plan and non-resorbable Donati’s stiches for the cutaneous plan after obtaining negative culture results and good healing.

**Table 1.** Patients’ characteristics.

	Patient 1	Patient 2	Patient 3	Patient 4	Patient 5
Sex	M	M	M	M	M
Age (years)	71	56	44	44	57
Etiology for HF	Idiopathic dilated CMP	Idiopathic dilated CMP	Non-compaction and valvular CMP	Ischemic CMP	Ischemic CMP
Indication of LVAD implantation	Destination therapy	Bridge to transplantation	Bridge to transplantation	Bridge to transplantation	Bridge to transplantation
Symptoms of DLI	Serosanguinous discharge Cutaneous erythema Pain on driveline parietal trajectory	Purulent discharge Cutaneous erythema Pain on driveline parietal trajectory	Pain on driveline parietal trajectory	Purulent discharge Cutaneous erythema Pain on driveline parietal trajectory	Purulent discharge
Diagnostic imaging	US-CT	CT	CT	CT	CT
Pathogens	MSSA Achromobacter Xylodisans	MSSA	MSSA	MSSA	MSSA
Duration of NPWT (day)	5	13	11	9	16
NPWT re-dressing x times	1	2	1	1	2
WBCs (/mm^3^) before incision/before wound closure	20,400/8100	12,200/7200	11,000/6100	15,800/9500	11,200/7000
CRP (mg/L) before incision/before wound closure	194/28	280/45	83/6	43/6	94/5
Antibiotherapy	Cefepime-Vancomycin-iv-3 days Flucloxacillin-iv-9 days Rifampicin-Ciprofloxacin-po-14 days Co-trimoxazole-po-lifelong	Imipenem-Vancomycin-iv-4 days Flucloxacillin -iv-Rifampicin-po-4 days Daptomycine-iv-6 weeks	Cefepime iv-Clindamycin po-12 days Clindamycin po-6 weeks	Vancomycin iv-Tazobac iv-Clindamycin po 3 weeks Doxycycline po 6 weeks	Vancomycin-Co-Amox 4 days Flucloxacillin-Rifampicin iv 6 weeks
Follow-up after NPWT ablation (month)	25	1 (until heart tx)	13 (until heart tx)	6	8

## Outcomes

All patients were discharged in good condition, without signs of infection. Two patients underwent successful heart transplantation after 1 and 13 months. During transplantation, no sign of residual DLI was observed. The other patients (two on transplant waiting list and one with destination therapy) did not show any residual or repetitive infection during the follow-up within 6, 8, and 25 months.

## Standard institutional surgical protocol for LVAD driveline infection

Aqueous chlorhexidine solution used as an alternative local antiseptic method to Betadine in order to prevent the driveline’s discoloration. Following abscess drainage and infected tissues debridement, mechanical cleaning was done with chlorhexidine, hydrogen peroxide, and physiologic sodium chloride solutions. At the end of the procedure, NPWT was applied.

## Discussion

Complications following LVAD implantation can be observed on over 50% of implanted patients with twice more re-hospitalization compared to the patients without complications.^6^ The most frequent complications following LVAD implantation are driveline related infections, gastrointestinal bleeding, and stroke.^6^ Driveline infections are mostly seen during the first 3 months post-implantation, as well as much later than that as observed recently on our patients. Multiple studies show hospitalizing patients with DLI are an economic burden since they require not only frequent re-hospitalization but also an increased hospital length of stay.^5^ There is a significant correlation between increased body mass index and DLI.^7^

Although treatment of DLI is the main topic of our manuscript, prevention is also important to decrease the frequency of this complication. Implantation technique is crucial. Indeed, DLI frequency may double if driveline tunneling is above the fascia of rectus abdominis muscle^8^ and the externalization of only the silicone portion of the driveline considerably reduces the incidence of DLI.^9^

Diagnostic methods for DLI includes ultrasonography (USG), computed tomography, and positron emission tomography.^10^ The USG examination is easily accessible and a method that favors economy. We especially use this tool in the operating room in order to enhance our precision with incision and abscess location. Computed tomography is also an easily accessible method, and non-injected images could be used on the patients with renal failure. Kimura et al.^11^ published the results of Gadolinium-SPECT based diagnosis of DLI on 22 patients. No significant differences were noted in patient characteristics, wound appearance, or laboratory results. However, patients with positive Gadolinium uptake had a higher 1-year event rates. In addition to a positive skin culture at driveline exit site and short duration of antibiotic therapy, the uptake on Ga-SPECT-CT was a risk factor for surgical intervention (odds ratio 9.00; *p* = 0.018) and readmission (odds ratio 7.86; *p* = 0.0051).^11^

DLI treatment modalities include the use of antibiotics with local wound disinfectant dressing if the infection is localized only around the exit site.^5,10^ If the infection is profound and accompanied with an abscess, surgical drainage with debridement of infected tissues is necessary. Driveline relocation following surgical drainage and debridement is another technique proposed by some centers.

The NPWT is another treatment method used in addition to the surgical drainage and debridement.^12^ In this treatment, at the end of surgical intervention a sterile polyurethane foam sponge is placed into the wound cavity and covered by a thin adhesive film. An evacuation tube with fenestrations is then placed over a small opening in the film and sealed in place by another thin dressing to convert it to a closed wound. Obtaining a seal is imperative to create the vacuum for the wound.^13^ Additional pieces of the thin film may be placed around the site to create the seal. A specific pump generates sub-atmospheric pressure that can be adjusted from −75 to −125 mmHg both continuously or intermittently. The dressing can remain on up to 3 or 5 days.^13^ The objective of the NPWT is to improve local aspect of the wound first to allow closure later. Indeed, the advantages of the NPWT is to decrease the size of the wound, drain the infectious material, reduce locale edema, hasten granulation tissue formation by increased fibroblast, and improve local capillary circulation. Furthermore, patients may be sent home with portable NPWT device until wound closure, which will eventually reduce the length of hospitalization.

Recurrent infections as well as resistant patients to above mentioned treatments may require the usage of the muscle or omental flap techniques. Muscle or omental flap techniques can also be used on the patients who have tissue defects following large surgical debridement.^14^ Device ablation and implantation of a new device is the most radical but not the simplest way to treat driveline infection. Although in most cases removing LVAD is not necessary, this option can be used for the patients who had deep tissue, pump, or graft infections accompanied with DLI.^5,15^

## Limitations

Our article presents our institutional experience with NPWT based only on five patients who had driveline infection following HeartMate III^®^ implantation with no comparison group. Two patients had the chance to have a heart-transplantation within 1 and 13 months following NPWT, however that does not permit us to see long term effects of this treatment.

## Conclusion

The complications related to LVAD will be seen more and more in the future based on increase usage of these devices for end-stage heart failure treatment and especially with increased numbers for destination therapy. As proportionally, driveline infections following LVAD implantation keeps an important percentage compared to other complications and their management remains a challenge. The result of our NPWT method shows a successful and less invasive approach therapy.
